# Diagnostic, Predictive, and Prognostic Biomarkers in Non-Small Cell Lung Cancer (NSCLC) Management

**DOI:** 10.3390/jpm11111102

**Published:** 2021-10-27

**Authors:** Maja Šutić, Ana Vukić, Jurica Baranašić, Asta Försti, Feđa Džubur, Miroslav Samaržija, Marko Jakopović, Luka Brčić, Jelena Knežević

**Affiliations:** 1 Division of Molecular Medicine, Ruđer Bošković Institute, 10000 Zagreb, Croatia; Maja.Sutic@irb.hr (M.Š.); ana.vukic259@gmail.com (A.V.); Jurica.Baranasic@irb.hr (J.B.); 2 Hopp Children’s Cancer Center (KiTZ), 69120 Heidelberg, Germany; a.foersti@kitz-heidelberg.de; 3 Division of Pediatric Neurooncology, German Cancer Research Center (DKFZ), 69120 Heidelberg, Germany; 4 German Cancer Consortium (DKTK), 69120 Heidelberg, Germany; fedja1104@gmail.com (F.D.); miroslav.samarzija@gmail.com (M.S.); marko.jakopovic@kbc-zagreb.hr (M.J.); 5 Clinical Department for Respiratory Diseases Jordanovac, University Hospital Centre Zagreb, School of Medicine, University of Zagreb, 10000 Zagreb, Croatia; 6 Diagnostic and Research Institute of Pathology, Medical University of Graz, 8010 Graz, Austria; luka.brcic@medunigraz.at; 7 Faculties for Dental Medicine and Health, University of Osijek, 31000 Osijek, Croatia

**Keywords:** lung cancer, adenocarcinoma, squamous cell lung cancer, biomarker, diagnosis, prognosis, targeted therapy, immunotherapy

## Abstract

Lung cancer is the leading cause of cancer-related deaths worldwide. Despite growing efforts for its early detection by screening populations at risk, the majority of lung cancer patients are still diagnosed in an advanced stage. The management of lung cancer has dramatically improved in the last decade and is no longer based on the “one-fits-all” paradigm or the general histological classification of non-small cell versus small cell lung cancer. Emerging options of targeted therapies and immunotherapies have shifted the management of lung cancer to a more personalized treatment approach, significantly influencing the clinical course and outcome of the disease. Molecular biomarkers have emerged as valuable tools in the prognosis and prediction of therapy response. In this review, we discuss the relevant biomarkers used in the clinical management of lung tumors, from diagnosis to prognosis. We also discuss promising new biomarkers, focusing on non-small cell lung cancer as the most abundant type of lung cancer.

## 1. Introduction

Lung cancer (LC) is the leading cause of cancer-related mortality worldwide, responsible for 18.4% of all cancer deaths. The latest GLOBOCAN database estimates that 2.2 million new lung cancer cases emerged in 2020 worldwide [1]. The American Cancer Society estimated the appearance of 230,000 newly diagnosed lung carcinoma cases in 2020 in the United States of America, with an anticipated mortality of 22–23% of the total cancer deaths [2]. Traditionally, lung cancer is classified into two major groups—non-small cell lung cancer (NSCLC; 80–85% of LC cases) and small cell lung cancer (SCLC; 15–20% of LC cases). The most common histologic subtype of NSCLC is adenocarcinoma (ADC), with an incidence of 40% [3]. Squamous cell lung carcinoma (SQC) comprises 20–30% of all LC cases [3]. Unfortunately, only a small proportion of NSCLC patients (<20%) are diagnosed at the early stage of the disease, while the tumor is localized and does not involve regional lymph nodes. However, the majority of NSCLC patients (47%) are still diagnosed at later stages (stages III/IV), when the tumor has already spread to multiple lymph nodes and/or to distant organs [4], which consequently impacts the median survival time that barely exceeds 18 months [5,6]. The 5-year relative survival for patients diagnosed at an advanced stage is approximately 6%, compared to patients diagnosed at early stages that are expected to surpass this survival timeframe in 61% of cases [4]. It is also important to note that the 5-year survival rate varies across countries. Japan has the highest 5-year survival rate of 30%, most likely due to a higher relative proportion of EGFR mutation-positive patients and efforts to improve personalized cancer care [7]. Most countries, including 21 countries from Europe, have a 5-year survival rate of 10–19%, while the lowest 5-year survival rate was recorded in India, Brazil, Thailand, and Bulgaria (<10%) [8].

### 1.1. Lung Cancer Classification

**Figure 1:**
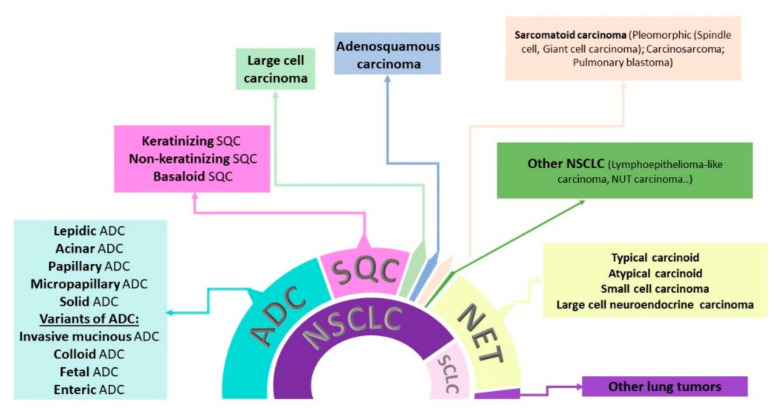
Classification of lung tumors based on resection specimens. The inner circle represents the traditional classification of lung tumors into non-small cell lung cancer (NSCLC) and small cell lung cancer (SCLC). The outer circle represents the WHO 2021 classification of lung tumors, in which SCLC is grouped with other types in the category of neuroendocrine tumors. ADC—adenocarcinoma; SQC—squamous cell carcinoma; NET—neuroendocrine tumors.

Accurate histologic classification is crucial in the management of lung tumors because it is known that some therapeutics have potentially harmful side effects (such as bevacizumab [9]) or are inefficient (such as pemetrexed [10]) for the treatment of SQC. Until recently, guidelines were lacking for more specific sub-classifications of lung tumors from small biopsies and cytological samples. The World Health Organization Classification of Lung Tumors of 2015 addressed this issue and incorporated relevant genetic and immunohistochemical (IHC) aspects of different tumor subtypes [11]. Major revisions in the approach to adenocarcinoma, based on the 2011 IASLC/ATS/ERS Classification of lung adenocarcinoma [12], were accepted in the 2015 and 2021 WHO classification [13]. Furthermore, diagnosis of large cell carcinoma was restricted to the resected tumors lacking morphological and immunohistochemical signs of differentiation. SQC tumors are now classified as keratinizing, non-keratinizing, and basaloid. Lymphoepithelial carcinoma is also included in the SQC type. SCLC is a member of the neuroendocrine carcinomas, under neuroendocrine neoplasms of the lung. The group of sarcomatoid carcinomas comprises pleomorphic carcinoma (encompassing spindle cell and giant cell carcinoma), pulmonary blastoma, and carcinosarcoma. It is clearly stated that, in small biopsy and cytology samples, diagnoses of large cell and adenosquamous carcinoma should not be made, but in these cases, not-otherwise-specified NSCLC should be used [13]. The summary of this classification is shown in Figure 1.

### 1.2. Diagnosis of Lung Cancer

The initial evaluation of patients with susceptibility to LC is usually made with a chest X-ray, CT scan, and/or PET-CT scan [14]. Due to differences in management options for various LC subtypes, the accurate identification of specific histologic subtypes needs to be performed on tissue samples collected in various ways, such as bronchoscopy, transbronchial needle aspiration, transthoracic fine-needle aspiration, core biopsy, among others [15]. The general differentiation between LC subtypes is based on the morphological features of tumor samples stained with hematoxylin and eosin. Tumors with morphological evidence of keratinization or intercellular bridges are classified as SQC, while tumors with mucin production or glandular architecture are classified as ADCs [16]. However, morphology can be insufficient for the proper classification of tumor types in some cases, especially when the tumor is poorly differentiated or when it lacks specific morphologic or phenotypic features [17]. In this case, it is recommended to use immunohistochemistry for the classification of LC, but saving enough tumor tissue for predictive biomarker testing [11,18].

It is also worth mentioning that, in addition to tumor tissues, liquid biopsy, relatively recently, has became a valuable source of material for diagnostic purposes, not only of lung cancer, but also of many other types of cancer. This arose from a paradoxical situation: advances in technology and the accumulation of the new knowledge has brought us into a position in which we need to obtain significant amounts of samples for multiple analyses of a growing number of different biomarkers, with minimally invasive approaches. The problem is that traditionally obtained cytological samples are often insufficient for comprehensive molecular examinations, leading us to need new tissue sources. In principle, liquid biopsy is defined as the sampling of the non-solid biological materials/tissues. Liquid biopsy is any tumor-derived material circulating through the blood or any other body fluid. The most frequently studied, or used, materials from the blood, in NSCLC diagnosis, are circulating tumor cells (CTCs) or circulating tumor DNA (ctDNA). It has also been shown that exosomes, which contain RNAs derived from the patient’s tumor, can be found in the blood of patients [19,20]. Later, in this article, it will be explained in more detail how micro RNAs (miRNAs), single-stranded noncoding RNAs, could be used as a potential diagnostic biomarker in the management of NSCLC.

### 1.3. Treatment Options

The successful treatment of LC depends on several important factors: stage at diagnosis (defined with tumor size, regional lymph nodes involvement, and the presence of metastasis), histologic subtype, and molecular characterization. The therapy of non-metastatic NSCLC usually consists of surgical resection, adjuvant and neoadjuvant chemotherapy, as well as immunotherapy in the unresectable stage III of NSCLC [21]. The successful treatment of LC in the advanced stages depends on the histologic subtype, the presence of targetable mutations, and the patient’s clinical status and comorbidities [15]. The approval of targeted therapeutics, such as epidermal growth factor receptor (EGFR)—tyrosine kinase inhibitors (TKIs) [22,23] or anaplastic lymphoma kinase (ALK) inhibitors [24], improved survival in patients positive for these driving somatic mutations. Similar benefits for patients’ survival were shown with immune checkpoint inhibitors that target programmed-death 1 receptor (PD-1) or its ligand (PD-L1) [25,26]. However, most NSCLC patients are EGFR or ALK negative [27], and less than one-third of advanced lung tumors express PD-L1 in more than 50% of tumor cells [26]. Finally, patients not eligible for targeted or immune therapy are treated with platinum-based chemotherapy as the first line, and eligible patients will receive it after the failure of targeted or immune therapy [15,28]. We would also like to mention that a relatively new strategy in NSCLC treatment is to combine different approaches, including targeted therapy, immunotherapy, radiotherapy, and chemotherapy. The fact that the tumors develop resistance to any form of therapeutic strategy has forced us to look at the problem from a different angle. There are numerous different examples of combined therapy approaches. For example, in 2018, The FDA approved the addition of the PD-1 inhibitor pembrolizumab to platinum-based chemotherapy. The results of this study showed improved outcomes in patients with squamous NSCLC of any level of PD-L1 expression, when compared with the use of chemotherapy treatment only (KEYNOTE-407/NCT02775435) [29]. Another example is the CheckMate 012 study that combined nivolumab with erlotinib in patients with advanced, EGFR-mutant NSCLC, who were EGFR tyrosine kinase inhibitor (TKI)–naive or TKI-treated, but had not received chemotherapy. Previous studies in mouse models have reported that activation of the oncogenic EGFR pathway enhances the susceptibility of lung cancer to PD-1 blockade, suggesting that the combination of PD1 blockade with EGFR TKIs may be a promising therapeutic strategy. The results of this study revealed that treatment with nivolumab and erlotinib was tolerable, with durable responses in patients with EGFR-mutant, TKI-treated NSCLC [30]. Trials, such as this one, will definitely improve the treatment of lung cancer. However, the search for predictive biomarkers is the only option to lead us to better treatment strategies.

In this review, we discuss well-defined biomarkers for the management of patients with NSCLC, including diagnostic, predictive, and prognostic biomarkers. We also point to some novel and exciting molecular biomarkers that have not yet been included in clinical practice, but show potential for translation to the clinics in the future. The key definitions for the biomarkers are summarized in Box 1.

Box 1 **A summary of the key definitions.**

Biomarkers are measurable characteristics that indicate biological processes of patients or their tumors or can indicate responses to treatment intervention [31]. **Diagnostic biomarkers** should be able to detect and differentiate specific diseases from other conditions or identify a relevant subtype of a particular disease [32]. **Predictive biomarkers** are used to identify individuals most likely to benefit from certain treatments [31]. **Prognostic biomarkers** can indicate the likelihood of a clinical outcome or the pace of disease recurrence and progression [6]. The most informative characteristics of biomarkers are specificity and sensitivity. **Sensitivity** is a percentage of true positive cases in the analyzed group of patients, and **specificity** is a percentage of truly negative cases in the control group [31].

## 2. Diagnostic Biomarkers Used in NSCLC Clinical Management

Approved drugs for patients with NSCLCs are especially beneficial for patients with ADCs carrying driver alterations due to the higher rate of targetable mutations present compared to SQCs [31]. The delineation of the histology is therefore essential for optimal treatment decisions. In this section, we discuss the biomarkers used in daily clinical practice, such as immunohistochemical and blood/serum diagnostic biomarkers. We also point to novel biomarkers that have not yet been included in routine clinical practice, but show promising diagnostic potential.

### 2.1. Immunohistochemical Biomarkers

The primary technique for the diagnosis and classification of lung cancer histology in clinical practice is immunohistochemistry (IHC). Based on the recent publication about best practice recommendations for the usage of IHC in lung cancer diagnostic, TTF-1 (Thyroid Transcription Factor 1) (for ADC) and p40 (for SQC) are designated as the best markers for the subtyping of NSCLC, especially when the 8G7G3/1 monoclonal antibody is used for TTF-1 detection. Napsin A is the second best marker for ADC, while p63 can be positive both in lung ADC and some other tumors. However, while for the TTF-1 only the focal positive nuclear reaction is considered as a valid positive, for p40 more than 50% of tumor cells must demonstrate nuclear positivity [32]. In the case of a TTF-1 negative ADC, one should always think about the possibility of metastasis and apply additional IHC. Rare lung tumors and undifferentiated neoplasms always require additional sets of antibodies. As discussed above, in the case of clinical suspicion of a primary lung tumor and a negative reaction with p40/TTF-1, one should diagnose the tumor as a non-small cell carcinoma-not otherwise specified (NSCC-NOS) and send it for additional testing for predictive biomarkers, without “wasting” tumor tissue for definitive diagnosis/classification [3].

### 2.2. Circulating Tumor Protein Biomarkers from the Blood and Serum

In comparison to IHC, which requires tumor samples obtained by biopsy or resection, blood/serum samples are more easily obtained and a helpful tool in clinical settings. Cytokeratin 19 fragment (CYFRA 21-1), carcinoembryonic antigen (CEA), squamous cell carcinoma antigen (SCCA), and carbohydrate antigen 125 (CA125) are well established and the most commonly used blood/serum biomarkers for the detection of LC, used either as a single biomarker [33] or in panels of several combined biomarkers [34,35]. Several studies propose different combinations of well-established biomarkers and novel markers, such as cancer/testis antigen 1B (CTAG1B/NY-ESO-1) [36], prolactin (PRL) [37], retinol binding protein (RBP), 1-antitrypsin (ATT) [38], thymidine kinase 1 (TK1), neuron specific enolase (NSE) [39], or autoantibodies Annexin A1-Ab and α-enolase-Ab (ENO1) [40]. All mentioned studies report that specificity and sensitivity of assays are increased with the addition of novel markers compared to the performance of a single marker. Testing autoantibodies together with established biomarkers could also increase usefulness of established biomarkers in early cancer detection, as autoantibodies are produced early in tumorigenesis and can be detected in the serum sooner than tumor-associated antigens [41,42].

### 2.3. miRNAs—Potential Diagnostic Biomarkers

Micro RNAs (miRNAs) are single-stranded noncoding RNAs, 20–25 nucleotides in length that can alter gene expression post-transcriptionally through direct degradation of mRNA or repression of translation. They have an important role in numerous biological processes, including cell growth, apoptosis, inflammation, and cancer [43]. Since miRNAs are stable and can be detected in various biological fluids, such as in serum, plasma, pleural fluid, urine, or cerebrospinal fluid, miRNAs could be ideal non-invasive diagnostic biomarkers [44].

Expression levels of certain miRNAs vary between pathological conditions and healthy controls, and these differences might enable new strategies in the diagnosis of many diseases, including LC. For example, miR-33a-5p and miR-128-3p are down-regulated in LC tissue compared to adjacent normal tissue and a combination of these miRNAs shows good diagnostic characteristics [45]. Some miRNAs are shown to be NSCLC subtype-specific, such as miR-205 for squamous cell LC [46,47] and miR-375 for adenocarcinoma [48]. Additionally, miR-93 and miR-221 have increased expression in squamous cell lung cancer compared to adjacent non-malignant tissue, while high levels of miR-100 are correlated with adenocarcinoma in smokers [49]. A miRview lung assay was developed for expression analysis of eight miRNAs (miR-106a, miR-125a-5p, miR-129-3p, miR-205, miR-21, miR-29b, miR-375, and miR-7). Based on the miRNA expression profile, the assay can differentiate between SCLC and NSCLC, as well as SCLC from carcinoid lung tumor or squamous from non-squamous NSCLC with high accuracy, showing a great diagnostic potential [50].

Interestingly, several studies indicated that miRNAs could also be used as circulating diagnostic biomarkers of early stage NSCLC. It has been shown that miR-324-3p is significantly up-regulated, while miR-1285 was significantly down regulated, in plasma samples of patients with stage 1 squamous cell LC, compared to healthy controls [51]. Wang et al. identified a panel of five serum miRNA for NSCLC diagnosis in patients of different races. The panel can discriminate NSCLC from controls and differentiate between malignant lesions and benign nodules. The panel includes miR-483-5p, miR-193a-3p, miR-25, miR-214, and miR-7. In both testing and validation cohorts, these miRNAs were significantly elevated in NSCLC compared to controls [52].

Sozzi et al. investigated the diagnostic potential of the miRNA signature classifier (MSC) assay on plasma samples collected within the Multicentric Italian Lung Detection (MILD) trial that included patients with low-dose computed tomography (LDCT) screening results. The authors used an expression ratio of 24 miRNAs and reported promising assay characteristics for LC detection. The LDCT alone showed similar sensitivity as the MSC assay, but the reported false–positive rate was high (19.4%). However, when used in combination with the miRNA expression ratio, the LDCT false–positive ratio was reduced to 3.7% [53]. Montani et al. also proposed miR-Test as a first-line screening tool [54].

Based on the literature, it seems that only the combination of several miRNAs will significantly raise diagnostic accuracy. However, there are some limitations and inconsistencies in the reported studies. For example, there is only a partial concordance in the miRNAs repertoire used in different studies. Moreover, only a few panels were validated on bigger cohorts [53,54], which implies the need for further validations. Furthermore, the type of samples used for diagnosis (plasma vs. serum) and a good normalization control for the RT-qPCR approach are still not well defined. To translate miRNAs into routine clinical practice, scientists should first agree on solving the aforementioned inconsistencies and establish models that could be validated in large-scale clinical trials.

## 3. Predictive Biomarkers in NSCLC Management

The histologic type of NSCLC is still used as a predictive factor for chemotherapy treatment. For instance, pemetrexed treatment was demonstrated as beneficial for patients with non-squamous NSCLC, while patients with squamous NSCLC had a similar overall survival (OS) in both pemetrexed and placebo groups. Therefore, non-squamous histology is a predictive factor for pemetrexed-based chemotherapy [55]. In addition to histology, specific genetic alterations are also predictive biomarkers for NSCLC treatment. At present, there are several predictive genetic biomarkers used in clinical settings, which will be described in this section, together with promising new biomarkers.

**Figure 2:**
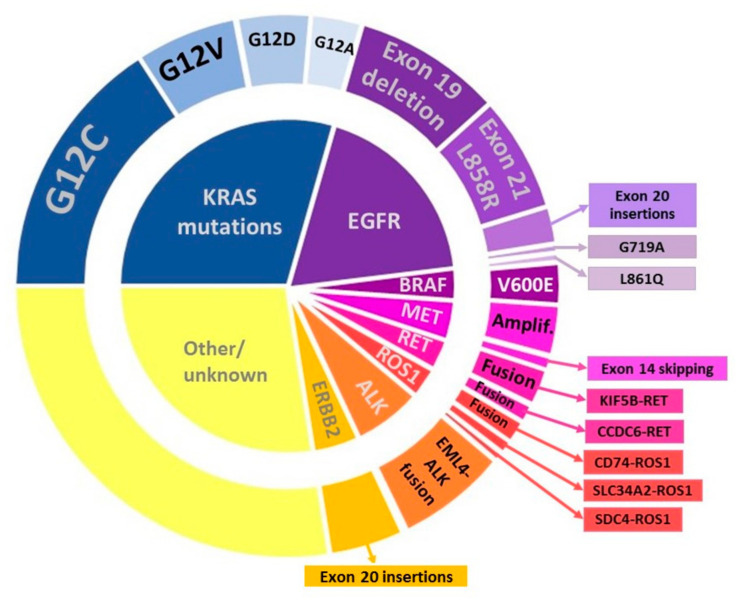
Molecular alterations in lung adenocarcinoma.

The identification of the predictive markers has a great impact on treatment choice. Therefore, the detection of the known genetic alterations is a prerequisite for treatment. When testing for predictive biomarkers, two important factors need to be considered: obtaining an adequate specimen and choosing the right method of testing [56,57]. One of the problems in biomarker testing is tissue exhaustion due to series of single-gene tests for assessing multiple types of genetic alterations [56]. The College of American Pathologists (CAP), the International Association for the Study of Lung Cancer (IASLC), and the Association for Molecular Pathology (AMP) issued updated guidelines for LC testing in 2018. These guidelines recommend routine multigene testing of all advanced NSCLC with an adenocarcinoma component for EGFR mutations and ALK and ROS1 rearrangements, together with additional genes (RET, MET, Her2, KRAS, and BRAF). They also recommended to test samples of SQC in younger patients (<50 years of age) and who have never smoked. Testing for T790M is recommended in all patients with sensitizing EGFR mutations who have progressed after treatment with EGFR-TKIs [58]. The current Pan-Asian guidelines recommend the testing mentioned above and PD-L1 immunohistochemistry to be performed in all patients with advanced non-squamous NSCLC [59]. Some local guidelines, such as those in Austria, recommend reflex testing for all non-squamous carcinoma regardless of the stage, using multigene testing and reflex testing for PD-L1 in both SQC and AC [60]. In this section, we focus on the most commonly used biomarkers that predict response to available targeted therapies and immunotherapy. Molecular alterations used as predictive biomarkers are summarized in Figure 2. Approved targeted therapeutics are summarized in Table 1. Additionally, we discuss new predictive biomarkers reported in the literature that might become relevant for routine use in the future.

### 3.1. Predictive Biomarkers for Targeted Therapy in NSCLC Patients

Epidermal growth factor receptor (EGFR): also known as HER1, it is a member of the protein kinase superfamily. The activated EGFR is involved in different biological processes, such as cell proliferation, differentiation, and apoptosis [61]. However, specific EGFR mutations, also known as activating or sensitizing mutations, can cause constitutive activation of the receptor, leading to uncontrolled cell division and tumor pathogenesis. These mutations are more common in ADC female, non-smoking patients from East Asia [62]. The two most common EGFR mutations are exon 19 deletion (45–50% of all mutations) and exon 21 (L858R) substitution (35–45% of all mutations) [62]. Although these mutations are more common in ADCs, they do appear in SQC as well, but at a lower rate (3.3% in Western and 4.6% in Asian populations). The aforementioned EGFR mutations are predictive for their response to drugs called tyrosine kinase inhibitors (TKIs) that bind to tyrosine kinase receptors, reversibly or irreversibly, and inhibit downstream signaling [63]. To date, the US Food and Drug Administration (FDA) has approved five TKIs for the treatment of NSCLC. Erlotinib and gefitinib are first-generation reversible tyrosine kinase inhibitors that have been proven beneficial to ADC patients [64,65]. Afatinib is a second-generation TKI that binds to EGFR and other members of the ERBB family irreversibly [66]. Osimertinib is a third-generation EGFR TKI, used to treat patients harboring EGFR T790M mutation, the main cause of drug resistance to the first-generation TKIs [67].

Anaplastic Lymphoma Kinase (ALK): the ALK gene encodes an enzymatic protein known as ALK tyrosine kinase receptor or CD246 [68], a member of the insulin receptor superfamily of tyrosine kinases. ALK activation leads to the activation of downstream signaling pathways, such as PI3K/AKT, RAS/MAPK, and JAK/STAT [69]. Approximately 2–7% of NSCLC patients have alterations in the ALK gene. ALK alterations include rearrangements, amplifications, and point mutations [70]. These alterations can cause constitutive expression and activation of the ALK protein, leading to oncogenic phenotype and tumor pathogenesis [69]. The most common rearrangement is an echinoderm microtubule-associated protein-like 4 (EML4) inversion rearrangement with ALK, resulting in an EML4–ALK fusion oncogene [5,70]. Many different variants of the EML4–ALK fusion oncogene have been described, as well as rearrangements of ALK with other genes, such as KIF5B, KLC1, TFG, TPR, HIP1, STRN, DCTN1, SQSTM1, NPM1, BCL11A, and BIRC6 [70]. ALK rearrangements are more common in younger, ADC patients who have never smoked with a median age of 55 years [70,71]. ALK rearrangements are almost always mutually exclusive with EGFR and KRAS mutations [71]. The first drug to be approved for ALK rearrangements was crizotinib, an inhibitor of ALK and ROS1 [72]. However, patients tend to develop resistance to the treatment due to either a new ALK point mutation (like L1196M) or as a result of the activation of EGFR or KRAS signaling pathways [71]. Therefore, new drugs were developed for patients resistant to crizotinib, such as ceritinib, alectinib, brigatinib, and lorlatinib [5].

ROS Proto-Oncogene 1, Receptor Tyrosine Kinase (ROS1): ROS1 is a tyrosine kinase receptor, involved in cell growth and differentiation [73]. Although it is mainly expressed during embryonic development, ROS1 is also expressed in limited amounts in adults, especially in lung tissue. In 1–3% of lung adenocarcinomas, rearrangements of the ROS1 gene are observed [74]. These rearrangements cause the constitutive activation of the ROS1 gene, leading to cell proliferation, survival, and migration. Many ROS1 fusion partners have been identified, and CD74–ROS1 is the most commonly found in NSCLC [73]. In a similar manner to ALK, ROS1 rearrangements are more commonly found in younger patients who have never smoked, and are almost exclusively observed in ADCs [74,75]. Crizotinib is also approved for ROS-rearranged NSCLC [76], but resistance can occur as in treatment of ALK rearrangements. Many studies and trials are currently testing the effectiveness of multi-kinase inhibitors, such as ceritinib [77], brigatinib [78], alectinib [79], cabozantinib [80,81], and lorlatinib [82], in the treatment of ROS1-rearranged NSCLC [73].

V-raf murine sarcoma viral oncogene homolog B (BRAF): the BRAF gene encodes a cytosolic serine/threonine protein kinase B-Raf, a member of the Raf kinase family. It is downstream of KRAS (Kirsten rat sarcoma oncogene) and is involved in the mitogen-activated protein kinase (MAPK) signaling pathway [83]. The constitutive activation of the mutated BRAF gene activates the MAPK signaling pathway, leading to increased cell proliferation and cell growth. These mutations are observed in 2–4% of NSCLC, primarily in ADC [83]. A V600E mutation is the most common and occurs in approximately 50% of BRAF-positive NSCLC cases. V600E constitutively activates BRAF, which then phosphorylates the downstream effector MEK [84,85,86]. It seems that V600E mutation is mutually exclusive with KRAS mutations, while BRAF non-V600 positive patients might harbor KRAS mutations as well [83]. Non-V600 mutations might be associated with patients with a smoking history and of male gender [84,85,86]. The FDA approved a combination of dabrafenib (BRAF inhibitor) and trametinib (MEK inhibitor) to treat NSCLC patients with advanced or metastatic tumor carrying BRAF V600E mutation.

Mesenchymal–epithelial transition tyrosine kinase receptor (MET): upon ligand binding, MET mediates the activation of several signaling pathways, such as PI3K/AKT, STAT3, SRC/FAK, and MAPK/ERK. Reported MET mutations in NSCLC include amplification, an exon 14-skipping mutation, and mutations in the kinase domain [87]. It is reported that NSCLC patients harboring MET exon 14-skipping mutation and MET overexpression have a better response to crizotinib [88] and tivantinib [89,90]. The FDA also approved capmatinib for patients with metastatic NSCLC that harbor the MET exon 14-skipping mutation, based on results from the GEOMETRY mono-1 trial [91]. Most recently, in 2021, the FDA approved tepotinib for metastatic NSCLC with MET exon 14 skipping alterations (VISION trial, NCT02864992).

Rearranged During Transfection (RET): RET fusions are found in 1–2% of NSCLC, mainly in ADC [92,93]. The most common fusion partner of RET is KIF5B [94]. Coiled-coil domains of RET fusion partner proteins foster dimerization of RET fusion proteins, leading to the constitutive activation of RET kinase [27] and the activation of several kinases, including MAPK, (PI3K)/AKT, and JNK [93]. Various trials and case reports have shown the benefit of cabozantinib [93,95] and vandetanib [96] treatment in RET-rearranged NSCLC patients. In 2020. The FDA approved selpercatinib and pralestinib for the treatment of NSCLS with RET gene alterations.

Neurotrophic receptor tyrosine kinase (NTRK): NTRKs are involved in the regulation, growth, differentiation, and programmed cell death of neurons in both peripheral and central nervous system. Upon binding with respective ligands, they activate different downstream signaling pathways, such as the Ras/Raf/MAPK pathway, PI3K/Akt/mTOR pathway, and PLCγ/PKC pathway [97]. Most studied NTRK1 fusions are MPRIP–NTRK1 and CD74–NTRK1, found in 3% of lung ADCs [98]. TRIM24–NTRK2 fusion has also been found in some lung adenocarcinomas [99]. The FDA approved larotrectinib (pan-NTRK inhibitor) for the treatment of solid tumors that have NTRK gene fusions based on the LOXO-TRK-14001 (NCT02122913), SCOUT (NCT02637687), and NAVIGATE (NCT02576431) clinical trials. The FDA also approved entrectinib for the treatment of ROS1-positive and NTRK-positive solid tumors based on the ALKA, STARTRK-1 (NCT02097810), and STARTRK-2 (NCT02568267) clinical trials. Some other multi-kinase inhibitors are being tested in ongoing trials, such as cabozantinib, which is in a phase II study (NCT01639508), and MGCD516, which is in phase I/Ib (NCT02219711).

Neuregulin-1 (NRG1): NRGs are growth factors for the ErbB family of receptor tyrosine kinases. NRG1 is the most studied member of the NRGs group and is a ligand of HER3/4 [100]. Its primary role is in normal physiology during neural development [101], but it can also have a pathologic role in several types of cancer, including NSCLC. Fusions of NRG1 with several identified partners (to date), of which the CD74–NRG1 fusion is the most common, are relatively rare. The estimated incidence of NRG1 fusions in NSCLC is 0,3% [102]. Afatinib, an inhibitor of ErbB receptors, is a treatment option for NSCLC patients harboring NRG1 fusions [103,104,105]. An ongoing phase I/II trial study (NCT02912949) is evaluating the activity and safety of Zenocutuzumab (MCLA-128), an anti-HER2/3 antibody, in patients with solid tumors, including NSCLC, harboring NRG1 fusion. Another ongoing phase 2 trial study, CRESTONE (NCT04383210), is investigating Seribantumab, an anti-HER3 monoclonal antibody, for treatment of NRG1 fusion-positive advanced/metastatic solid tumors [106]. The summary of currently approved and available therapies is shown in Table 1.

### 3.2. Predictive Biomarkers for Immunotherapy in NSCLC Patients

The immune evasion of cancer cells is considered as a hallmark of cancer [107]. It is well established that tumor cells can express or produce immune-suppressive molecules that inhibit the function of T lymphocytes, which helps them to evade immune surveillance. One of the known immune evasion mechanisms that cancer cells exploit is through immune-inhibitory pathways called immune checkpoints. Immune checkpoints are proteins expressed on the surface of immune cells that recognize the corresponding ligand and transmit stimulatory or inhibitory signals that modulate immune response [108]. In this Section, we will discuss the programmed death receptor1 (PD-1) and its ligand (PD-L1) in the context of NSCLC immunotherapy.

The programmed death-ligand 1 (PD-L1) protein, also called B7-H1, is encoded by the CD274 gene. PD-L1 is constitutively expressed on the surface of many immune cells, such as macrophages, antigen-presenting cells, B cells, and T lymphocytes. PD-L1 binds to a programmed death receptor (PD-1) predominantly expressed on the surface of activated cytotoxic T cells. This binding leads to the suppression of the immune system and is important in preventing an autoimmune response during inflammation [109]. However, PD-L1 is also expressed by many different tumor cells, including lung cancer, and its expression enables their evasion from immune response [110]. Higher expression of PD-L1, at both mRNA and protein level, was observed in NSCLC compared to healthy lung tissue, regardless of NSCLC type [111]. Currently, four FDA-approved monoclonal antibodies targeting the PD-1/PD-L1 interaction are used for the treatment of patients with NSCLC: nivolumab, pembrolizumab, atezolizumab, and durvalumab [112]. Summary of immune checkpoint inhibitors approved by FDA and EMA is shown in Table 2. However, the validity of PD-L1 expression as a predictive biomarker is questionable, because it is has been shown that patients with low PD-L1 expression, less than 1%, responded exceptionally to anti-PD-1/PD-L1 treatment. It is possible that PD-L1 expression changes over time and is inconsistent throughout the tumor tissue. Furthermore, there are different clones of PD-L1 antibodies, with different cut-off points in the immunohistochemical analysis of PD-L1 expression, and they are not identical [113].

### 3.3. Novel Predictive Biomarkers for Targeted Therapy

Even though current biomarkers notably improved NSCLC treatment, there is still an ongoing need for novel predictors and targeted therapeutics that could help to achieve better outcomes and cost-effectiveness in treating patients with NSCLC, especially those with a squamous subtype diagnosis. In this Section, we summarize the literature for reported potential biomarkers that are already being tested in several clinical trials (summarized in Table 3) and might become relevant in the future.

Kirsten rat sarcoma viral oncogene homolog (KRAS): the KRAS gene encodes for RAS protein, a GTPase crucial for the activation of several pathways, including the Raf/MEK/ERK, PI3K/AKT/mTOR, and RalGDS/RalAIB pathways [6]. Mutations in KRAS cause the constitutive activation of KRAS signaling, leading to cell proliferation and survival. Activating KRAS mutations are almost exclusive to ADCs and are more frequent in Western populations (~30% in Western and ~10% in Asian populations), making them the most common mutations in Western NSCLC cases [114]. Considering the mutual exclusivity of KRAS and EGFR mutations, as well as the downstream role of RAS proteins in the EGFR signaling pathway, KRAS status could be used to determine whether a certain patient would benefit from an EGFR inhibitor treatment [115]. There are also several ongoing clinical trials investigating therapeutics for KRAS G12C mutation, the most common KRAS mutation in NSCLC.

Fibroblast growth factor receptor 1 (FGFR1): Fibroblast growth factor receptor 1 (FGFR1) is a cell surface tyrosine kinase involved in the regulation of proliferation, differentiation, cell migration, and survival [116]. FGFR1 amplification, mutations, and rearrangements can cause the constitutive activation of the receptor and contribute to tumor promotion [117]. Amplification of FGFR1 is identified in 9–20% of SQCs and up to 15% in lung ADCs, and seems to be more common in male patients with a smoking history [118,119]. It has been shown that the aberrant expression of FGF or FGFR family reduces the sensitivity of mesenchymal-like NSCLC cells to EGFR inhibitors [120]. Numerous nonselective FGFR inhibitors were evaluated in NSCLCs over the years, such as dovitinib [121], lenvatinib [122], pazopanib [123], nintedanib [124], brivanib [125], ponatinib [126], lucitanib [127], and regorafenib [128]. Unfortunately, most of the studies observed limited antitumor activity and high drug toxicity. However, assessing the validity of FGFR as a predictive biomarker is still an ongoing endeavor, and the list of novel FGFR inhibitors is still expanding [129].

Discoidin domain receptor 2 (DDR2): Discoidin domain receptors, DDR1 and DDR2, are tyrosine kinases involved in mammary gland development, long bone growth, and the occurrence of many types of diseases, including arthritis, atherosclerosis, and cancer [130]. The deregulation of DDR pathways, due to somatic mutations or the altered expression of receptors, can cause tumor growth and promote cell migration and invasion [131]. Mutations in the DDR2 gene are observed in 2–4% of lung SQCs and approximately 30% of SQC cases have elevated levels of the DDR2 protein [132,133,134]. Currently, clinical activity of multi-kinase inhibitor MGCD516 is being evaluated in NSCLCs and head and neck cancer populations with DDR2 mutations and/or other activating mutations (MET, NTRK2, NTRK3), rearrangements (MET, RET, AXL, NTRK1, or NTRK3), or amplifications (MET or KIT/PDGFRA/KDR) (NCT02219711).

Human epidermal growth factor receptor 2 (HER2/ERBB2): HER2 is an important member of the epidermal growth factor receptor family (ERBB) involved in the activation of PI3K-AKT and MEK-ERK proliferation pathways. HER2 is activated by dimerization with other ERBB family receptors, ligand-activated EGFR and HER3, or homodimerization when it is overexpressed. This usually happens in cancer, which leads to increased cell proliferation and the promotion of cell cycle progression. Other types of HER2 aberrations found in cancer include gene amplification and mutations [135,136,137]. The overexpression of the HER2 protein is observed in 2–38% of lung ADCs and in 1–16% of SQCs [138,139,140]. Mutations in the HER2 gene are found in approximately 2% of NSCLCs, and the most common is the exon 20 HER2 in-frame insertion. These mutations are more frequently observed in female patients who have never smoked [141]. HER2 amplification was detected in 10–20% of lung ADCs [140].

**Table 3:** Summary of ongoing clinical trials for novel predictive biomarkers for targeted therapy.

Gene	Alteration	Drug	Eligible Patients	Trial Name	Treatment	Ref.
KRAS	G12C	AMG 510 (Sotorasib)	Previously treated, locally advanced, unresectable, or metastatic NSCLC	CodeBreak 200 NCT04303780 Phase 3	AMG 510 vs. Docetaxel	[142]
		MRTX849 (Adagrasib)	Previously treated for metastatic NSCLC	KRYSTAL-12 NCT04685135 Phase 3	MRTX849 vs. Docetaxel	[143]
	KRAS mutation in codons 12 or 13	Selumetinib + Docetaxel	Locally advanced or metastatic NSCLC	SELECT-1 NCT01933932 Phase 3	Selumetinib + Docetaxel vs. Placebo + Docetaxel	[144]
		Abemaciclib (LY2835219)	Stage IV NSCLC patients who have progressed after platinum-based chemotherapy	JUNIPER NCT02152631 Phase 3	Abemaciclib vs. Erlotinib	[145]
	G12V, G12C	Carboplatin + paclitaxel + bevacizumab	IIIB or stage IV NSCLC patients eligible for platinum-based chemotherapy and are chemotherapy naïve	NCT02743923 Phase 3	Carboplatin-paclitaxel-bevacizumab vs. Cisplatin-pemetrexed	[146]
FGFR1	FGFR1 amplification (>5 copies)	Dovitinib	Pretreated advanced SQC	NCT01861197 Phase 2	Dovitinib	[121]
	Aberrant signaling	E7080 + Carboplatin + Paclitaxel	Advanced or metastatic NSCLC	NCT0083281 9Phase 1	E7080 + Carboplatin+ Paclitaxel	[122]
		Pazopanib	Resectable stage I/II NSCLC	NCT0036767 9Phase 2	Pazopanib	[123]
		Nintedanib BIBF 1120	Stage IIIB/IV or recurrent NSCLC after the failure of first-fine chemotherapy	LUME-Lung 1 NCT00805194 Phase 3	Nintedanib + Docetaxel vs. Placebo + Docetaxel	[124]
DDR2	DDR2 mutations	MGCD516	Advanced solid tumor, including NSCLC	NCT02219711 Phase 1	MGCD516	
HER2	exon 20 mutations	Afatinib	Pretreated patients with advanced NSCLC	NICHE NCT02369484 Phase 2	Afatinib	[147]
		Pyrotinib	Advanced non-squamous NSCLC patients who failed platinum-based chemotherapy	PYRAMID-1 NCT04447118 Phase 3	Pyrotinib vs. Docetaxel	
		Pertuzumab+ Trastuzumab + Docetaxel	NSCLC patients harboring HER2 exon 20 mutation or insertion	NCT03845270 Phase 2	Pertuzumab+ Trastuzumab + Docetaxel	
	HER2 mutations	Neratinib, temsirolimus	Advanced (stage IIIB) or metastatic (stage IV) NSCLC	NCT01827267 Phase 2	Neratinib or Neratinib + Temsirolimus	[148]
	HER2 mutations or overexpression	Trastuzumab deruxtecan (DS-8201a)	Unresectable and/or metastatic NSCLC	DESTINY-Lung01 NCT03505710 Phase 2	Trastuzumab deruxtecan (DS-8201a)	[149]

### 3.4. Novel Predictive Biomarkers for Immunotherapy

In this Section, we summarize the literature for reported predictive biomarkers that are already being tested in several clinical trials (e.g., dMMR, MSI, or TMB). We also report potentially novel biomarkers that are reported by only a few studies, but which we believe might become relevant to the clinics in the future. Ongoing clinical trials for novel immunotherapy biomarkers are summarized in Table 4.

Deficient mismatch repair (dMMR) and microsatellite instability (MSI): DNA mismatch repair system (MMR) is a highly conserved repair mechanism in cellular evolution. The MMR system maintains integrity and stability of the genome by overlooking genetic recombination and repairing the identified mismatched nucleotides while avoiding deletions or insertions of DNA microsatellites [150]. Deficiency in the MMR system (dMMR) is caused by germline mutations or in the case of the occurrence of sporadic tumors, most commonly due to epigenetic alterations, such as the methylation status of the four key genes MLH1, MSH2, MSH6, and PMS2 [151], which are active as DNA MMR enzymes in heterodimeric form, usually as MLH1/PMS2 and MSH2/MSH6. In the dMMR status, one or more of the MMR proteins are dysfunctional or not expressed [150]. That leads to genetic hypermutability most frequently at the sites of microsatellites, the so called microsatellite instability (MSI) [152]. Standard sites in testing panels for MSI are BAT25, BAT26, D5S346, D2S123, and D17S250. A tumor is considered as MSI-H if alterations occur in two or more repeats [150]. MSI is not common in NSCLC; according to several studies, MSI frequency is <1% [152,153].

Tumor mutational burden (TMB): TMB is defined as the total number of non-synonymous mutations present in a tumor. TMB could be used as a predictive biomarker for nivolumab (PD-1 targeted antibody) and ipilimumab (CTLA-4 targeted antibody) treatment [154]. NSCLC patients with higher TMB (TMB-H, ≥10 mutations per megabase) treated with a combination of nivolumab and ipilimumab showed a significantly longer progression-free survival (PFS), compared to patients receiving chemotherapy as a first-line treatment. Furthermore, TMB and PD-L1 expression were shown to be independent biomarkers [155].

Interferon-gamma (IFNγ): interferon-gamma is a cytokine with diverse roles in the innate and adaptive immune system. IFNγ plays a role in antiviral activity, antimicrobial activity, and antitumor activity [156]. It was reported in the literature that patients treated with anti-PD-L1 antibodies (such as durvalumab [157] or nivolumab [158]), with a higher expression of IFNγ, had longer progression-free and overall survival, compared to patients with a lower IFNγ expression.

Tumor infiltration lymphocytes (TILs): tumor infiltration lymphocytes (TILs) are immune cells that are present in tumors. Since some of them have a role in tumor progression and some in tumor regression, they are an important target in the evaluation for anti-cancer therapy [159]. Fumet et al. observed that, in NSCLS patients treated with nivolumab, a high expression of CD8+ TILs was significantly associated with a better response rate (RR) and progression-free survival (PFS) [159]. In patients treated with EGFR TKIs, a CD8+/CD4+ ratio could be a predictive response to immunotherapy. A lower ratio is indicative of a lower response rate, compared to a higher ratio [160].

T-cell immunoglobulin and mucin-domain containing-3 (TIM-3): T-cell immunoglobulin and mucin-domain containing-3 is a member of the TIM family of immune-regulatory proteins. TIM-3 is expressed by many immune cells, and it is being studied as a therapeutic target that likely modulates immune response [161]. Limagne et al. showed that a high level of TIM-3 expression in peripheral lymphoid cells after the initiation of nivolumab treatment is an important factor that negatively affects the response to anti-PD-1 therapy. Progressive patients had greater TIM-3 expression than stable and responding patients [162].

## 4. Prognostic Biomarkers

### 4.1. Prognostic Biomarkers Used in NSCLC Clinical Management

Prognostic biomarkers indicate the likelihood of a patient’s clinical outcome, most commonly defined as overall survival, progression-free survival (PFS), or disease-free survival rate [32]. Identifying prognostic markers in lung tumor patients is important, because it allows the recognition of patient subpopulations that might anticipate different outcomes or might benefit from different types of therapies [163]. Unlike predictive markers that interact with the treatment to influence the outcome, it is not expected that the treatment effects will be different when patients groups are distinguished by prognostic markers alone [164]. The most reliable prognostic markers are reported on patients samples involved in large studies or placebo-controlled trials, because patient characteristics in cohorts are better defined and uniform [163]. Moreover, to enlarge patient cohorts and increase statistical power, scientists also use cost-effective ways to find potential prognostic markers with a meta-analysis of comparable trials or studies.

Prognostic markers can be genes, mRNA, proteins, or miRNAs. The most studied is protein expression, usually evaluated with immunohistochemistry. Advancements in technology, such as mass spectrometry, also influence a growing number of studies focusing on proteomic signature [165]. Similarly, tumor profiling, using microarrays or next-generation sequencing, generates new potential prognostic signatures based on the mRNA [166,167,168,169], methylation [170,171], or miRNA [172] status. There is no doubt that molecular tumor profiling is a very promising and productive research area that has arisen in the last decade, with numerous emerging biomarkers reported to date. However, despite the enormous amount of data available on molecular biomarkers, results are often not reproducible, partially due to the heterogeneity of study designs, techniques used, and interpretation of the data. Therefore, many molecular prognostic markers, to date, have not managed to pave their way in routine clinical use. In addition to molecular biomarkers, there are routinely used biomarkers for prognosis assessments that are well established in clinical settings, such as TNM stage, patient age, gender, and performance status. TNM stage, an internationally accepted classification system, uses tumor size (Tis-T4), nodal involvement (N0-N3), and the presence of distant metastasis (M0-M1c) to characterize the extent of the disease. Stage groups are defined based on different combinations of T, N, and M components [173]. LC stages correlate well with survival—for example, 90% of lung cancer patients diagnosed at the early stage, when tumor has not spread to surrounding lymph nodes, are predicted to reach the 5 year survival estimation, while only 12% of patients diagnosed at the advanced stage are expected to survive that long [174]. Regarding metastasis, a single metastatic spot is not as detrimental as multiple metastatic sites [164]. Sometimes TNM staging is combined with the molecular testing of the tumor to guide prognostic assessment and treatment. Performance status (PS) is the assessment of patients’ functionality level and their ability of self-care. Oncologists assess performance status with different tools, including the ECOG (Eastern Cooperative Oncology Group) performance status or KPS (Karnofsky performance status). The KPS scoring relies on a scale that ranges from 0 to 100%, in which 100% indicates no evidence of disease or symptoms and 0% indicates death. The ECOG scoring system, also called the World Health Organization (WHO) performance status, assesses performance status using a 5-point scoring system. A PS score 0 indicates normal activity and ability to function without restraints, while a score 5 indicates death [175]. The ECOG PS is often used as eligibility criteria for clinical trials, such as chemotherapy or immune therapy trials, for which the required PS is often 1 or 0.

### 4.2. Novel Prognostic Molecular Biomarkers

In this Section, we summarize the available literature for reported novel prognostic biomarkers (Table 5). Although some proposed novel prognostic biomarkers are still controversial, due to inconsistencies among reported studies, we believe that they show good potential and they might become relevant with time as the number of validation studies increases.

TP53: TP53 gene encodes the tumor-suppressor protein p53, an important player in cell cycle regulation, senescence, autophagy, apoptosis, and DNA repair in response to damaging agents [176]. Mutations in p53 lead to a loss of p53 tumor-suppressor functions, resulting in excessive cell proliferation and cancer promotion [177]. In NSCLCs, it seems that mutations of p53 are more frequent in SQCs compared to ADCs (77% vs. 47%, respectively) [178]. To date, several studies reported that the p53 mutational status in NSCLCs is associated with poorer survival and increased resistance to cancer therapy, compared to TP53^WT^ [178,179,180]. However, some studies did not confirm p53 as a prognostic factor in NSCLCs [181].

Vascular endothelial growth factor (VEGF): tumor cells supply nutrients to grow and disseminate via existing blood vessels or angiogenesis. The vascular endothelial growth factor (VEGF) affects microvascular permeability, stimulates the growth of endothelial cells, and is pre-eminent in the formation of a new blood vessel in angiogenesis. VEGF overexpression is associated with tumor recurrence and metastasis, and is common in many cancer types, including lung cancer [182]. Several studies report that overexpression of the VEGF indicates poor prognosis in NSCLCs [183,184,185].

Class III β-tubulin (TUBB3): TUBB3 is an isotype of beta-tubulin that is normally found in various tissues [186,187,188], where polymers of tubulin form microtubules. Several studies indicate that high expression of TUBB3 is an indicator of poor prognosis in NSCLCs [189] and correlate an abundant TUBB3 expression with a reduced response to anti-tubulin-based chemotherapy, such as taxane or vinorelbine [190,191,192].

Ki-67: Ki-67 is encoded by the MKI67 gene. Since it is expressed in actively dividing cells throughout the cell cycle, reaching its expression peaks at the M phase, Ki-67 serves as a good proliferation marker [193]. The high expression of Ki-67 has been correlated with poor prognosis in several cancer types, including NSCLCs [194,195,196].

Excision repair cross complementing group 1 (ERCC1): ERCC1 protein plays an important role in the nucleotide excision repair pathway (NER) that is important for the maintenance of genomic stability. Studies indicate that a low expression of ERCC1 is an indicator of poor survival and that expression is generally higher in SQC histology [197,198].

Transforming growth factor-beta (TGF-β): TGF-β belongs to the cytokine family and is involved in cell proliferation, differentiation, and extracellular matrix production [199]. Although there is very little information on its prognostic potential, a few studies reported that a high TGF-β1 protein expression indicates poor prognosis [200,201]. Further investigations are needed to confirm these findings, but current studies on this issue are currently lacking.

Lymphocyte-activation gene 3 (LAG-3): lymphocyte-activation gene 3 is expressed on Tregs, and is involved in mediating their function [202]. It has been shown that NSCLC patients whose TILs were LAG-3^−^ have longer recurrence-free survival (RFS) and OS versus NSCLC patients whose TILs were LAG-3^+^. Moreover, a high expression of LAG-3 is correlated with a higher expression of PD-1 on TILs. When taking both LAG-3 and PD-L1 expression into account, patients whose tumor cells are PD-L1^−^ and LAG-3^−^ TILs have longer RFS than patients who are PD-L1^+^ or LAG-3^+^ or both positive [203]. However, Hald et al. have shown that the expression of LAG-3 on TILs in primary NSCLC tumors and metastatic lymph nodes is associated with improved survival [204], so further validation studies on its use as potential prognostic biomarker are needed.

KIAA1522: even though the KIAA1522′s function is still unknown, in vitro experiments have shown that it is involved in the oncogenic KRAS signaling in lung cancer cells. In NSCLC patients, a lower OS has been linked with a high expression of KIAA1522, compared to those with a low expression of the protein, regardless of the stage and histological type (SQC and ADC). Furthermore, patients with a lower KIAA1522 expression that were treated with platinum-based chemotherapy have longer OS, in comparison to those with a lower KIAA1522 expression treated platinum-based chemotherapy [205].

Neutrophil-to-lymphocyte ratio (NLR) and platelet-to-lymphocyte ratio (PLR): inflammation plays an important role in both the development and propagation of lung cancer. Pretreatment neutrophil-to-lymphocyte ratio (NLR), as well as platelet-to-lymphocyte ratio (PLR), are signs of systemic inflammatory response, and they are closely related to the prognosis of various cancers [206]. Recently, there have been many reports of NLR and/or PLR as prognostic markers for various treatments. Studies show that, in patients with metastatic NSCLC, treatment with nivolumab elevated pretreatment levels of NLR and PLR, which are associated with a worse OS and a lower response rate (RR) [207,208].

**Table 5:** Summary of novel prognostic biomarkers.

Prognostic Biomarker	Alteration	Outcome
TP53	p53 mutations	Poorer survival, increased resistance to therapy
VEGF	High expression	Poor prognosis, tumor recurrence, metastasis
TUBB3	High expression	Poor prognosis
Ki-67	High expression	Poor prognosis
ERCC1	Low expression	Poor prognosis
TGF-β	High expression	Poor prognosis
LAG-3	Low expression	Longer RFS and OS [203]
	High expression	Better survival [204]
KIAA1522	High expression	Lower OS
	High expression + platinum-based chemoterapy	Longer OS
NLR & PLR	High NLR and PLR + Nivolumab	Worse OS, lower RR

OR—overall survival; RFS—recurrence-free survival; RR—response rate.

## 5. Summary and Conclusions

Lung cancer is a complex disease, and its successful treatment depends on well-defined patient characteristics, histologic type of tumor, assessed biomarkers, and good and prompt communication between pathologists and oncologists. Over the last decade, significant progress in developing therapy with complementary predictive biomarkers for NSCLCs has been made. While diagnostic biomarkers are well established in clinical routine, the number of predictive biomarkers (and their associated therapeutical options) will increase in the near future due to the numerous research efforts to identify new potential biomarkers and the new trials that are incorporating these findings. However, how will those rapid changes affect routine clinical practice remains to be seen. Even though current biomarkers notably improved NSCLC treatment, there is still a need for novel predictors and targeted therapies that could help to achieve better outcomes and cost-effectiveness in treating patients with NSCLCs, especially those with SQC subtype.

## Figures and Tables

**Table 1:** Summary of targeted therapeutics approved for NSCLC treatment. The table was created based on the FDA and EMA database of approved therapeutics for the treatment of NSCLCs positive for genetic alterations. FDA—The Food and Drug Administration; EMA—European Medicines Agency.

Biomarker	Alteration	Targeted Therapy	Year of FDA Approval	Year of EMA Approval
EGFR	Exon 19 deletion	Erlotinib	2013	2011
		Gefitinib	2015	2009
		Afatinib	2013	2013
	Exon 21 (L858R) substitution mutation	Osimertinib	2018	2018
		Dacomitinib	2018	2019
		Erlotinib + Ramucirumab	2020	
	T790M	Osimertinib	2015	2016
	L861Q, G719X, S768I	Afatinib	2018	
ALK	ALK rearrangement	Crizotinib	2011	2012/2015
		Ceritinib	2014/2017	2015/2017
		Alectinib	2017	2017
		Brigatinib	2017	2018/2020
		Lorlatinib	2018	2019
ROS1	ROS1 rearrangement	Crizotinib	2016	
		Entrectinib	2019	
		Ceritinib	2019	
BRAF	V600E mutation	Dabrafenib + Trametinib	2017	
NTRK 1/2/3	Gene fusion	Larotrectinib	2018	2019
		Entrectinib	2019	2020
MET	Exon 14 skipping	Capmatinib	2020	
		Tepotinib	2021	
RET	RET rearrangement	Selpercatinib	2020	
		Praseltinib	2020	

**Table 2:** Summary of immune checkpoint inhibitors approved for NSCLC treatment. The table was created based on the FDA and EMA database of approved anti-PD-1/PD-L1 therapeutics for the treatment of NSCLCs. FDA—The Food and Drug Administration; EMA—European Medicines Agency; mNSCLC—metastatic non-small cell lung cancer; TPS-tumor proportional score.

Antibody	Target	Therapeutic Indication	Line of Therapy	Year of FDA Approval	Year of EMA Approval
Pembrolizumab	PD-1	Advanced/mNSCLC that express PD-L1 (TPS ≥ 1%)	Second-line	2015	2016
		mNSCLC with high PD-L1 expression (TPS ≥ 50%) ^1^	First-line	2016	2017
		Metastatic non-squamous NSCLC, regardless of PD-L1 expression	First-line (+ Carboplatin & Pemetrexed)	2017/2018	2018
		Metastatic SQC	First-line (+ Carboplatin and Paclitaxel or Nabpaclitaxel)	2018	2019
		Stage III NSCLC with PD-L1 expression (TPS ≥ 1%) ^2^	First-line	2019	
Nivolumab	PD-1	Advanced/mSQC	Second-line	2015	2015
		Advanced/mNSCLC ^3^	Second-line	2015	2016
		Recurrent/mNSCLC ^4^	First-line (+ Ipilimumab and 2 cycles of platinum-based chemotherapy)	2020	
		mNSCLC with PD-L1 expression (≥ 1%)	First-line	2020	
Atezolizumab	PD-L1	mNSCLC ^5^	Second-line	2016	2017
		Metastatic non-SQC NSCLC ^6^	First-line (+ Bevacizumab, Carboplatin, and Paclitaxel)	2018	2019
		Metastatic non-SQC NSCLC (PD-L1 ≥ 5%) ^7^	First-line (+ Nab-paclitaxel and Carboplatin)	2019	2019
		mNSCLC with high PD-L1 expression (≥ 50%) ^8^	First-line	2020	
Durvalumab	PD-L1	Stage III NSCLC ^9^	Maintenance therapy	2018	2018

^1^ Approved for mNSCLC with no EGFR or ALK genomic aberration and no prior systemic therapy. ^2^ Approved for patients with stage III NSCLC who are not candidates for surgical resection, definitive chemoradiation, or mNSCLC, with no EGFR or ALK genomic tumor aberrations. ^3^ Approved for progression on or after platinum-based chemotherapy. ^4^ Approved for recurrent/mNSCLC without ALK aberrations determined by the FDA approved PD-L1 IHC 28-8 pharmDx diagnostic device, approved for mNSCLS (PD-L1 ≥ 1%) with no EGFR or ALK genomic tumor aberrations. ^5^ Approved for mNSCLC, whose disease progressed during/after platinum-based chemotherapy. mNSCLC with EGFR or ALK genomic tumor aberration should receive atezolizumab only after the failing of the targeted therapy. ^6^ Approved for metastatic NSCLC, with no EGFR or ALK genomic tumor aberrations. ^7^ EMA approved as first-line therapy for metastatic NSCLC with PD-L1 expression of at least 5% FDA approval regardless of PD-L1 expression. ^8^ Approved for metastatic NSCLC with high PD-L1 expression (PD-L1 stained ≥ 50% of tumor cells) or PD-L1 stained tumor-infiltrating immune cells covering ≥ 10% of the tumor area, with no EGFR or ALK genomic tumor aberrations, determined by the FDA approved VENTANA PD-L1 (SP142) diagnostic assay. ^9^ Approved for patients with unresectable stage III NSCLC, whose disease has not progressed following concurrent platinum-based chemotherapy and radiation therapy.

**Table 4:** Summary of ongoing clinical trials for novel immunotherapy biomarkers.

Gene	Drug	Eligible Patients	Trial Name
dMMR & MSI-H	SL-279252 (PD1-Fc-OX40L)	MSI high and mismatch repair deficient NSCLC patients, excluding subjects with known EGFR sensitizing (activating) mutation or an ALK fusion	NCT03894618 Phase 1
	L-NMMA + Pembrolizumab	MSI high and mismatch repair deficient NSCLC patients	NCT03236935 Phase 1
TMB	L-NMMA + Pembrolizumab	Unresectable or metastatic tumor, TMB ≥ 10 mut/Mb	NCT03236935 Phase 1
	Atezolizumab + Bevacizumab	Stage IIIB or IV non-squamous NSCLC with TMB ≥ 10 mut/Mb	NCT03836066 (TELMA) Phase 2
TIM-3	TSR-022	NSCLC patients that have received no more than 2 prior lines of therapy, which must include a platinum-based chemotherapy and an anti-PD-(L)1 antibody	NCT02817633 (AMBER) Phase 1
TILs	LN-145	NSCLC patients that have received a single line of systemic therapy that included checkpoint inhibitor and chemotherapy with documented radiographic disease progression on or following this single line of systemic therapy	NCT04614103 Phase 2
	LN-145 + Pembrolizumab	Locally advanced or metastatic NSCLC with ≤ 3 prior lines of systemic therapy, excluding checkpoint inhibitors or ≤ 4 prior lines if 2 or more of the lines are TKI therapy	NCT03645928 Phase 2
	LN-145	Stage III or Stage IV NSCLC, who have previously received 1–3 lines of prior systemic therapy	NCT03645928 Phase 2
	LN-145 + Ipilimumab and Nivolumab	Stage III or Stage IV NSCLC who have previously received 1 line of approved checkpoint inhibitor monotherapy as the only prior line of systemic therapy	NCT03645928 Phase 2

## Data Availability

Not applicable.
